# Macroinvertebrate Assemblages along the Longitudinal Gradient of an Urban Palmiet River in Durban, South Africa

**DOI:** 10.3390/biology11050705

**Published:** 2022-05-05

**Authors:** Jeffrey Lebepe, Ntombifuthi Khumalo, Anele Mnguni, Sashin Pillay, Sphosakhe Mdluli

**Affiliations:** 1 School of Life Sciences, University of KwaZulu-Natal, Durban 4000, South Africa; khumalon799@gmail.com (N.K.); 213516754@stu.ukzn.ac.za (A.M.); sashinp94@gmail.com (S.P.); sphosakhemdluli@gmail.com (S.M.); 2 Department of Biology, School of Science and Technology, Sefako Makgatho Health Science University, Pretoria 0204, South Africa

**Keywords:** urban river, water pollution, anthropogenic litter, integrated habitat assessment score, Amphipoda, Notonemouridae

## Abstract

**Simple Summary:**

Rivers are the most threatened ecosystems globally. However, much attention has been directed to natural rivers as urban streams are regarded as unnatural with poor capacity to provide ecosystem services. This study was carried out to explore the capacity of an urban Palmiet River to provide refuge for aquatic biota and the importance of solid wastes in providing habitat for macroinvertebrates in severely disturbed urban rivers. The water was found to be of good quality in the upper reach/headwaters and polluted in the industrial area. However, the river showed the ability to self-purify as the water showed some improvement as it flows downstream. Various sensitive and tolerant groups of macroinvertebrates were found along the river. Although the current condition of the river can still support biodiversity and the functioning of the river, it is unclear if the system could endure further disturbance. Some sites with good-quality water showed low or absence of sensitive organisms due to poor habitat. It is evident that macroinvertebrate assemblages are influenced by the water quality and availability of physical habitat, and solid wastes may provide additional habitat in severely disturbed streams. These findings attest that urban river functioning does not differ from that of a natural river, and it should be appreciated as it provides important ecosystem services for local communities.

**Abstract:**

Urban rivers are regarded as unnatural because they drain catchments characterized by impervious surfaces. The present study explored macroinvertebrate communities in relation to water and habitat quality along the longitudinal gradient of an urban Palmiet River in Durban, South Africa. Sampling was conducted across six sites along the river. The water quality has shown a significant variation (ANOVA, *p* < 0.05) across six sites. Good-quality water was observed at Site 6, whereas Site 5 exhibiting hypertrophic condition. Sites 4 to 1 were all eutrophic; however, nutrient levels showed to decrease from Site 4 down to Site 2 and increased again at Site 1. A similar trend was observed for habitat quality, with Site 6 showing excellent and Site 5 exhibited poor habitat. Coinciding with water and habitat quality, macroinvertebrate diversity and abundance showed significant differences across six sites. Sensitive palaemonids, notonemourids, and amphipods were only observed in the headwaters and have contributed over 50% of the variation in abundance between Site 6 and other sites. The non-metric multidimensional scaling (NMDS) plot has also shown clear discrimination (MANOVA, *p* < 0.001) for the Average Score Per Taxon (ASPT) across the six sites. Macroinvertebrate communities have shown a clear association between water and habitat quality. These findings affirm the ecological importance of urban rivers as they provide refuge to aquatic biodiversity, with anthropogenic litter providing additional habitats for other taxa. Despite the current conditions supporting biodiversity and the functioning of the river, it is unclear if the system could endure further disturbance.

## 1. Introduction

Freshwaters are known to be biodiversity hotspots, with their species constituting about 10% of species on Earth [1]. However, freshwaters are the most threatened ecosystems as they serve as a repository for contaminants from the Earth’s surface. Other factors threatening freshwater functioning include over-abstraction of water, species invasion, climate change, and of particular interest, land-use change, which influences the hydrologic regime of the system [2,3]. In urban catchments, natural vegetation on land surfaces has been replaced by parking lots, roads, stormwater drains, and sidewalks which influences the hydrologic regime as they reduce infiltration of water into the ground and accelerate runoff to ditches and streams [4,5].

As a result, urban rivers experience an extremely high flow regime during rainy seasons, which ultimately destroy natural habitats and reduce nutrient retention [6,7]. High water velocity changes stream community structure along the river as it mobilizes bottom substrate and scours aquatic biotas that are susceptible to high flow [8,9]. However, anthropogenic litter restores heterogeneity of the river substrate resulting in increased habitat for aquatic biota [10]. Moreover, aquatic biotas are known for their adaptive strategies to different flow velocity, which influences their resilience in urban streams [11].

Despite urban rivers showing the ability to support aquatic biota, they still received less attention as they are regarded as a highly degraded system with poor capacity to provide ecosystem services [12]. Similar to natural rivers, urban rivers improve the livelihood of the nearby communities by providing a variety of ecosystem services such as flood control, soil erosion regulation, and water quality regulation [13,14]. Moreover, urban rivers provide recreational services such as canoeing, fishing, swimming, etc. [15]. Beyond ecological and recreational services, urban rivers may also attract business and leisure travelers due to their water-based features [16]. According to Reisinger et al. [4], the rate of ecosystem functions may be equivalent or even higher in urban streams than in agricultural or those draining forested catchments.

The Palmiet River is a unique urban stream characterized by highly variable stretches along the longitudinal gradient. The river passes through an urbanized residential area in the upper reach, followed by an industrial area in the middle reach, then a nature reserve and another urban residential area in the lower reach [13,17]. Although urban rivers are known for their already compromised physical habitat, they can still provide sanctuary to macroinvertebrates, with anthropogenic litter providing additional habitat [10,18]. In the present study, the following hypotheses were tested: (1) Macroinvertebrate communities would exhibit spatial variation along the longitudinal gradient of the Palmiet River, with a site within an industrial area showing lower taxa richness and abundance. (2) Macroinvertebrate assemblages would be strongly associated with the physical habitat and water quality. (3) The presence of anthropogenic solid waste materials would increase the abundance of some macroinvertebrates.

## 2. Materials and Methods

### 2.1. Study Area

**Figure 1:**
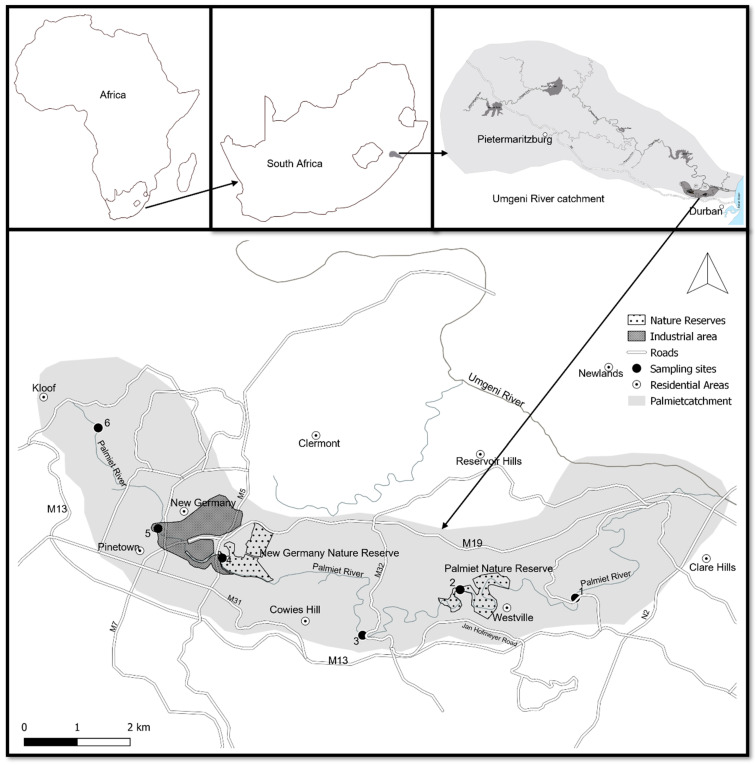
The Palmiet River catchment with sampling sites represented by black dots.

The Palmiet River originates from the Kloof escarpment and drains about 37 km^2^ catchment comprising urban residential and industrial areas with two community nature reserves, New Germany Nature Reserve and Palmiet Nature Reserve (Figure 1). The river feeds the Umgeni River, which is known as one of the largest river systems in KwaZulu-Natal (Figure 1) [17,19]. Sampling was conducted at six sites along the Palmiet River (Figure 1).

### 2.2. Water Sampling and Analysis

Sampling was carried out seasonally from April 2017 to February 2018 to cover different hydrologic flow regimes. However, due to urban rivers being sensitive to flash flood, we sampled four weeks after the flood during flooding seasons so that the macroinvertebrates could recover. Physical variables, i.e., temperature, pH, dissolved oxygen, salinity, and total dissolved solids, were measured in situ using Hanna multiparameter instrument (Model: HI98194). Three water samples were collected randomly in a 1 × 10 m transect using 1-L sampling bottles at each sampling site during each season. The river depth was mostly <0.5 m, so the water was sampled from approximately half the depth. Samples were kept in a cooler box filled with ice. The samples were later sent to an accredited laboratory for chemical analysis. In the laboratory, nitrite (NO_2_), nitrate (NO_3_), ammonia (NH_3_), potassium (K), sulfate (SO_4_), and phosphate (P) were measured. The guideline suggested by DWAF [20], WHO [21], and US-EPA [22] were used as the main set of criteria for water quality evaluation.

### 2.3. Habitat Assessment 

Macroinvertebrate habitat was evaluated were adapted from McMillan [23] (Appendix A). The components evaluated included stream habitats for macroinvertebrates (55%) and the physical characteristics of the river stretch (45%). Habitats were classified into three categories, stone in current (SIC), vegetations, and other habitats where the latter was comprised of stone out of current (SOC), bedrocks, gravel sand, and mud (GSM), and anthropogenic litter such as pipes, plastics, tins, etc. The weighting of these three habitats were 20%, 15%, and 20% for stone in current (SIC), vegetations, and other habitats, respectively. Components for stream physical condition included width, depth, biotopes make-up, water color, riparian vegetation availability, etc. (Appendix A). The Total Habitat score was calculated as the sum of final scores for each habitat category, whereas the Total IHAS score was the sum of Total Habitat (55%) and Stream Condition (45%) scores. Anthropogenic litter and their effect on water flow as stream characteristics were included under the Stream Condition. Anthropogenic litter was assigned a score of 0 where absent, 2 if similar items were observed, and 4 for a mixture of items. Anthropogenic effect on water flow was assigned a score of 2 if there was a severe effect (notable influence on water velocity) and 4 if there was no effect. Table 1 was used to classify the habitat condition.

### 2.4. Macroinvertebrate Sampling

Macroinvertebrate sampling was conducted seasonally from April 2017–February 2018 to cover different hydrologic regime. However, urban rivers are susceptible to flood [24], so sampling was carried out four weeks after the flood during flooding seasons. Sampling was carried out using a protocol adapted from Dickens and Graham [25], where different biotopes were sampled in each site. The biotopes were divided into aquatic vegetation, gravel, sand and mud, and stone biotope. Samples from marginal and aquatic vegetations were pulled together and treated as vegetation biotope. For the stone biotope, specimens collected from the bedrock, stone in and out of current were treated as one entity. Sampling was conducted for approximately 5 min in each biotope and 2 min for handpicking those that might have been missed by the sampling procedure. Macroinvertebrates were identified to the lowest taxonomic level possible using guides by Gerber and Gabriel [26], Gerber and Gabriel [27]. Some specimens were fixed in 70% ethanol for further identification and auditing in the laboratory. South African Scoring System (SASS5) scores were calculated during each sampling, and the Average Score Per Taxon (ASPT) was calculated to determine the ecological health of the river stretches.

### 2.5. Data Analysis

Data were analyzed using R version 4.1.1. Analysis of variance (ANOVA) was used to evaluate seasonal variation for water variables, habitat scores, Shannon-Weiner and Simpson’s Diversity indices, species richness, and abundance in each site and across six sites. The confidence interval for ANOVA was set at *p* < 0.05. The Package “ggplot2” [28] was used to ordinate the association between water quality, sites, habitat quality, and macroinvertebrates using Principal Component Analysis (PCA). Simper function was used to perform pairwise comparisons of sites to find out the average contribution for each taxon to the overall abundance dissimilarity. Non-Metric Multidimensional Scaling (NMDS) plot was used to visualize the ASPT across different sites in the Palmiet River. The dispersion was evaluated using the function “betadisper” and the significance was tested using adonis in the Vegan package with the confidence level set at *p* < 0.01. 

## 3. Results

### 3.1. Water Quality

**Figure 2:**
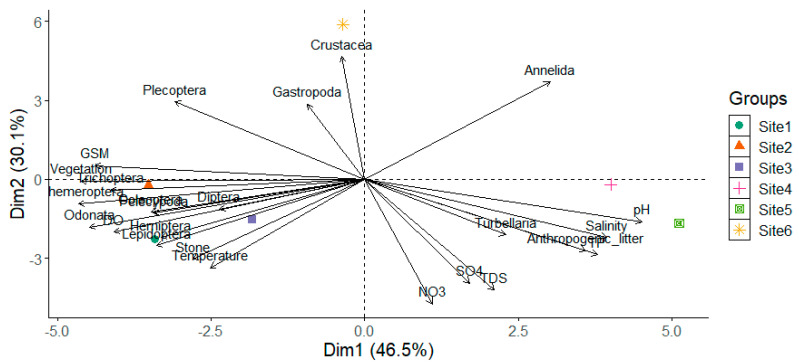
Principal Component Analysis ordination plot showing the association between environmental variables, habitat, taxa, and sampling sites.

Results for water variables are reported in Table 2. The water quality showed a great variability along the longitudinal gradient, with relatively lower levels of variables being observed at Site 6, which is located at the headwater. Neutral to alkaline pH was recorded through the study, with higher pH reported at Site 5 (Table 2, Figure 2). The pH has shown a significant difference across the sampling sites (ANOVA, F = 10.53, *p* < 0.05). The pairs of sites which showed significant differences were Site 1–Site 5, Site 4–Site 2, Site 5–Site 2, Site 3–Site 5, Site 6–Site 4, and Site 6–Site 5. A significant difference has been observed for temperature across the six sites (ANOVA, F = 56.97, *p* < 0.05) with dissolved oxygen showing no significant difference (ANOVA, F = 0.74, *p* > 0.05). A significantly higher level of TDS was observed at Site 5, with Site 6 showing lower TDS (ANOVA, F = 53.27, *p* < 0.05) (Table 2). Coinciding with TDS level, salinity exhibited a significantly higher level at Site 5 (Figure 2), with a lower level recorded at Site 6 (ANOVA, F = 10.52, *p* < 0.05) (Table 1). Nitrite was only recorded at PR5, with nitrate and ammonia also showing higher concentrations at Site 5 and lower levels being recorded at Site 6 (Table 2). Sulfate and phosphorus have also shown significantly higher concentrations in Site 5, with a lower level being observed at Site 6. Despite most variables showing significant differences among numerous sites, no seasonal variation was observed for all variables in each site. A strong association was observed for nitrate and TDS, with dissolved oxygen showing to be poorly associated with salinity, pH, and phosphorus (Figure 2).

**Table 2:** Water quality variable levels recorded at six sites in the Palmiet River (mean ± standard deviation). SI units are in mg/L unless specified otherwise. The table also presents guidelines stipulated by DWAF (1996) and (CCME (2012) for the protection of aquatic ecosystems.

Variables	Site 1	Site 2	Site 3	Site 4	Site 5	Site 6	TWQR
pH	7.95–8.32	7.29–8.21	7.86–8.32	8.43–8.91	8.78–9.11	7.57–8.20	6.5–9.0 (CCME 2012)
Temperature	26.64 ± 0.46	19.43 ± 0.81	25.04 ± 0.28	19.31 ± 1.08	18.65 ± 1.24	16.46 ± 1.75	-
DO	89.30 ± 4.45	91.53 ± 3.80	93.23 ± 4.15	88.21 ± 7.06	89.02 ± 3.05	89.43 ± 5.23	-
TDS	345.02 ± 36.70	213.99 ± 16.66	275.58 ± 26.49	336.32 ± 33.31	372.18 ± 22.43	119.21 ± 14.60	-
Salinity	0.20 ± 0.01	0.21 ± 0.01	0.19 ± 0.01	0.24 ± 0.01	0.27 ± 0.04	0.18 ± 0.02	-
NO_2_	<0.10	<0.10	<0.10	<0.10	0.52 ± 0.00	<0.10	0.06 (CCME 2012)
NO_3_	2.73 ± 0.77	2.42 ± 0.37	2.68 ± 0.88	2.77 ± 0.68	3.10 ± 0.64	0.42 ± 0.17	13 (CCME 2012)
NH_3_	0.22 ± 0.01	<0.10	<0.10	0.84 ± 0.02	11.5 ± 0.00	<0.10	0.007 (DWAF 1996)
N	2.95	2.42	2.68	2.77	15.12	0.42	-
SO_4_	31.35 ± 7.36	30.04 ± 7.05	21.32 ± 0.68	29.50 ± 4.46	39.40 ± 15.60	10.51 ± 1.90	-
Phosphate	0.11 ± 0.03	0.09 ± 0.02	0.17 ± 0.19	0.20 ± 0.07	0.23 ± 0.07	0.06 ± 0.02	0.1 (USEPA 1986)

### 3.2. Macroinvertebrates Habitat

Habitat scores have shown a significant difference across six sites (ANOVA, F = 213.95, *p* < 0.05), with only Sites 1 and 2 showing similar habitat conditions (*p* > 0.05). The lower habitat score was observed at Site 5 (52%) which is within the industrial area. Site 1 has shown a good quality habitat with an IHAS score of 72% being recorded. This river stretch has shown heterogeneity in vegetation biotope, from riparian to aquatic vegetation. The water depth was below 0.5 m with no anthropogenic modification on the channel. Moreover, stone biotopes exhibited a high heterogeneous habitat with high variability of stone sizes. There was a considerable length of gravel and sand habitat with dark mud characterized by dead leaves and tree logs. Site 2, which is in the Palmiet Nature Reserve, showed a similar river stretch (68%) to Site 1 with good quality for all biotopes. 

Site 3 was characterized by bedrocks and boulders with less vegetation compared to Sites 1 and 2. However, the water level was below 0.5 m with heterogeneous vegetation biotope. This site exhibited an IHAS score of 56%. Site 4 was dominated by gravel, sand, and mud with 2 riffles stretching for approximately 2 m with scattered fringing vegetations. The site has shown an IHAS score of 57%. Similarly, Site 5 exhibited scattered vegetation and stones. This site was characterized by a 0.5–1 m deep pool with opaque water and has shown a significantly lower IHAS score (52%) (*p* < 0.05). Located in the headwaters was Site 6, which was characterized by a narrow channel and predominantly sand and mud habitat, with scatted heterogeneous vegetation habitat, roots, and tree logs. This site exhibited relatively shallow and clear water. A considerably high IHAS score was observed in this site (76%). Using a habitat quality classification protocol (Table 1), Sites 1 and 2 were classified as good (B) with few modifications, Sites 3 and 4 as fair (C), Site 5 as poor (D), whereas Site 6 was almost natural (A). 

### 3.3. Macroinvertebrates

**Figure 3:**
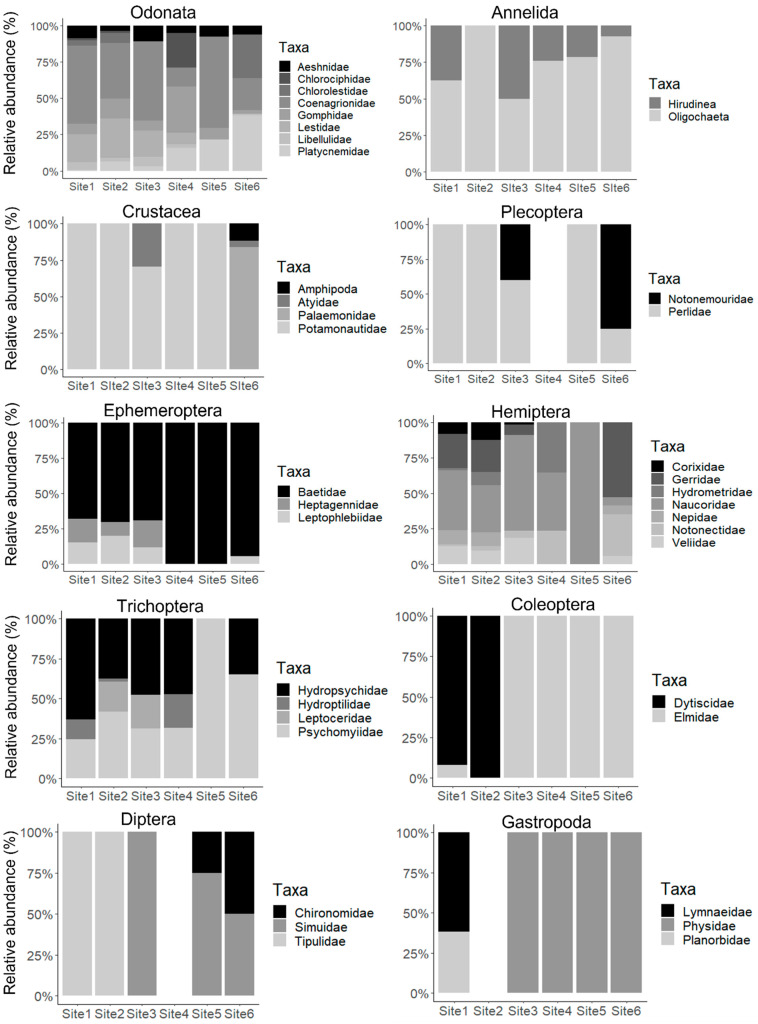
Taxa recorded in the Palmiet River during 2017 to 2018 surveys.

**Figure 4:**
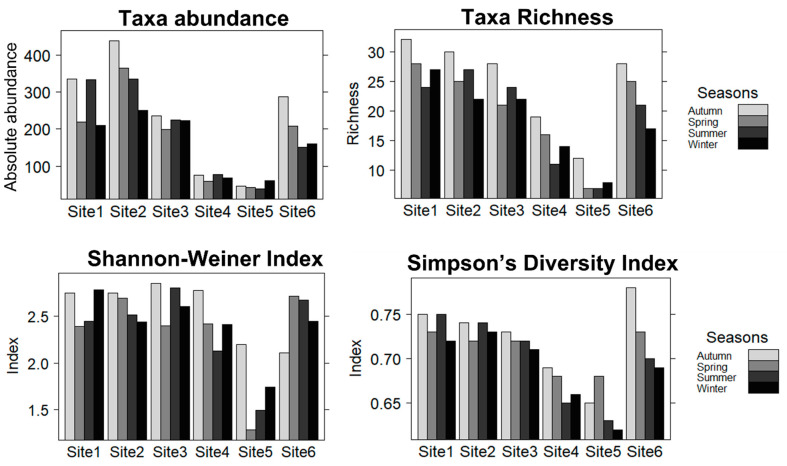
Taxa richness, abundance, Shannon-Weiner Index, and Simpson’s Diversity Index recorded along the longitudinal gradient of the Palmiet River in Durban.

Taxa recorded in this study are presented in Figure 3. Other taxa not presented in the figure include Planaria (Class: Turbellaria), Pyralidae (Class: Insecta, Order: Lepidoptera), and Corbiculidae (Class: Pelecypoda). Odonata was the most prominent in terms of taxa richness and abundance, with Coenagrionidae constituting 46% of the total abundance (Figure 3). Generally, a total number of 40 taxa were observed throughout the study, with Sites 1, 2, 3, and 6 showing significantly higher abundance compared to Sites 4 and 5 (ANOVA, F = 21.49, *p* < 0.05) (Figure 4). A notable absence observed was for amphipods, palaemonids, and atyids at Sites 1, 2, 4, and 5, and notonemourids, perlids, heptagenennids, leptophlebiids, chlorolestids, pyralids, corixids, gerrids, veliids, nepids, leptocerids, dytiscids, and elmids at Sites 4 and 5. Palaemonids were only recorded at Site 6 throughout the study (Figure 3). Oligochaetes, baetids, aeshnids, coenagrionids, gomphids, platycnemids, naucorids, and psychomyiids were observed across all six sites (Figure 3). Palaemonids, amphipods, hydropsyphids, and platycnemids constituted >50% of the abundance variation between Sites 5 and 6, whereas palaemonids, amphipods, hydropsyphids, lymnaeids, and gomphids constituted >50% of the abundance variation between Sites 4 and 6.

Sites 1, 2, and 6 showed high seasonal variations for taxa abundance, whereas taxa richness showed seasonal variations at all sites (Figure 4). Moreover, there was a significant difference for taxa richness across six sites (ANOVA, F = 18.23, *p* < 0.05), with lower richness being observed in Site 5. Similarly, a significantly lower taxa diversity was observed at Site 5 compared to all other sites (ANOVA, F = 7.02, *p* < 0.05) (Figure 4). Most taxa showed association with sites one, two, and three, which were characterized by good quality habitat, whereas crustaceans showed a strong association with Site 6 (Figure 2). Turbellaria was highly associated with sites 4 and 5, which were characterized by poor water quality and poor habitat accompanied by a large amount of anthropogenic litter (Figure 2). 

### 3.4. Average Score Per Taxon

**Figure 5:**
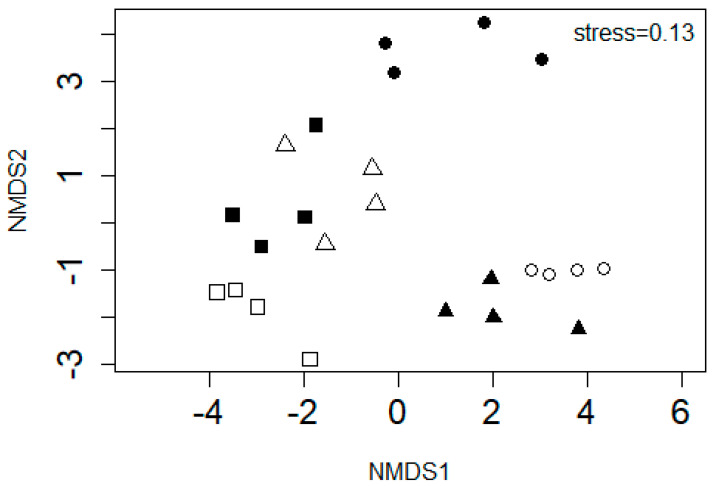
A non-metric multidimensional scaling plot ordinating the average score per taxon observed in Site 1 (□), Site 2 (▪), Site 3 (∆), Site 4 (▲), Site 5 (○), and Site 6 (●) in the Palmiet River.

Corroborating the species richness results was the SASS5 scores which showed clear distinction across the six sites (Figure 5). The mean ASPT of 6.14 ± 0.34, 6.35 ± 1.05, 7.02 ± 0.93, 5.56 ± 1.59, 4.47 ± 0.33 and 9.93 ± 1.14 were observed for Sites 1 to 6, respectively. The sensitivity score of the taxa observed across all six sites ranged from 1 to 8, with Sites 2 and 3 showing relatively high abundance for those with scores 7 and 8. The dispersion results were not significant (*p* < 0.001), with average distances to median being 2.82, 2.94, 2.83, 2.76, 2.14, and 3.06 for Sites 1 to 6, respectively. In contrast, significant results were observed for PERMANOVA (*p* < 0.001).

## 4. Discussion

### 4.1. Water Quality

The water quality has shown spatial variation along the longitudinal gradient of the Palmiet River. The headwaters of the river showed ideal water quality with pH, NO_2_, NO_3_, NH_3_, and P being within US-EPA [22], CCME [29], and DWAF [20] guidelines. The water quality in the headwaters is usually driven by natural characteristics such as rainfall intensity, frequency, and quantity, geology and soil type, river discharge, vegetation cover, and topography [30,31]. Site 5 is situated within the industrial areas, and it is the first point of entry for effluents into the Palmiet River. This site was characterized by an extremely high TDS and pH level and opaque water with a foul odor. This was also reflected in the nutrient level, which exhibited hypertrophic condition. Anthropogenic activities such as industries, wastewater discharge, and agriculture are known to be the primary drivers of nutrient enrichment in aquatic ecosystems [31,32]. It is evident that the industrial activities in the Pinetown area are affecting the water quality of the Palmiet River. 

However, the water quality improves as the river flows downstream, with Site 2 showing much improved conditions. In contrast, Site 1 showed another increase in water quality variables which suggests another pollution just outside Palmiet Nature Reserve. According to Tian et al. [33] and Šaulys et al. [34], rivers have the capacity to self-purify as a result of water attenuation between surface and ground waters, due to dilution as it is joined by good quality streams. Moreover, sedimentation, coagulation, and pollutants accumulation by aquatic biota may also reduce pollutant levels in the water column [35,36]. Nevertheless, self-purification capacity in urban rivers may be overwhelmed due to continuous pollution loading and irregular hydrologic regime of the water [37,38]. Other than industrial effluent discharge occurring at Site 5, the Palmiet River experience more sporadic bursts of wastewater system along the river due to blockages. Therefore, this may explain the variability of water quality along the Palmiet River. These results are comparable to those reported in other studies conducted in urban rivers [32,37,38].

Water quality parameters have shown no seasonal variation for all sites. Urban rivers are draining impervious surfaces, hence, susceptible to flash floods [4]. Therefore, sampling was carried out four weeks after the flood during flooding seasons which could be the explanation for the observed seasonal trend.

### 4.2. Habitat Quality

Urban streams are usually characterized by poor habitat due to constant disturbance induced by humans, resulting in channel modification and nutrient enrichment [39,40]. However, sites 1 and 2 have shown few modifications and were classified as good. Good quality habitat for sites 1 and 2 was expected as human activities are not permitted in these sites. Sites 1 was within the University of KwaZulu-Natal, whereas Site 2 was within the Palmiet Nature Reserve. Although Site 3 runs through a residential area, it was accessible to humans; hence, a few instances of anthropogenic litter were observed. Similarly, Sites 4 and 5 were also accessible, with some people dumping solid wastes in these sites. Site 5 has even shown opaque water with massive solid wastes, which have not influenced the water flow. In contrast, Site 4 exhibited a high accumulation of solid waste materials, which have even affected the water flow. Site 6 was located in the headwaters of the stream, and no modification was observed at this site. The site was characterized by a closed canopy with fast-flowing clear water. The observed habitat characteristics in the Palmiet River were comparable to those recorded in other urban rivers [10,41]. Moreover, channel modification, new types of sediment, and debris such as building rubbles and plastics, and other solids wastes are common in urban rivers [42,43]. 

### 4.3. Spatial and Temporal Variation of Macroinvertebrates

Macroinvertebrates are important components of lotic aquatic ecosystems, and they showed to respond rapidly to habitat destruction, change in hydrologic regime, and water quality deterioration [24,44,45]. Therefore, their community structures are regarded as good indicators of the ecological integrity of a river system. In the present study, Sites 1, 2, 3, and 6 exhibited higher abundance, richness, and diversity of macroinvertebrates. Sensitive taxa such as amphipods, palaemonids, and notonemourids were associated with Site 6, which was in its pristine state, and Site 3 which exhibited much-improved water quality compared to Sites 4 and 5. Taxa observed at Sites 4 and 5 were dominated by the less sensitive oligochaetes, leeches, potamonautids, coenagrionids, libellulids, planaria, psychomyiids, chironomids, to mention a few, which suggest water quality as the key driver of this trend. The trend was comparable to what was reported in other studies in both natural and urban rivers [45,46,47,48]. Chironomids and oligochaetes are tolerant taxa which are regarded as good indicators of pollution as they also strive in severely polluted waters [48,49].

Despite physico-chemical variables, physical habitat is known to influence macroinvertebrate assemblage [50]. Although natural habitat quality was poor at Sites 4 and 5, some taxa such as oligochaetes, psychomyiids, and chironomids were found within anthropogenic litter at these sites. It is evident that anthropogenic litter can also improve the heterogeneity of habitats in an aquatic system. Corroborating these results, Wilson et al. [10] recorded oligochaetes and larval stages of psychomyiids and chironomids within anthropogenic litter in an urban river. Moreover, similar taxa were observed within solid waste materials from the bottom of the near-shore part of a Włocławek Dam in Poland [18].

Sites 1 to 3 showed no substantial difference regarding taxa diversity, richness, and abundance. These sites were characterized by almost similar habitats and water quality. The most abundant taxa in these sites were coenagrionids, naucorids, lestids, baetids, and hydropsychidae, consecutively. No association was observed between the water quality and taxa diversity across these three sites. Contrastingly, a strong association was observed between taxa diversity and habitat scores. Coenagrionids and lestids inhabit emergent vegetation, beatids occupy rocky substrates, hydropsychidae inhabit decaying tree logs, whereas naucorids are found crawling on the bottom substrate [51]. These sites were characterized by dense vegetation, different-sized stones, and open gravel sand and mud biotope, which provided adequate habitats for these taxa.

Despite the spatial variation, Sites 1, 2, and 6 showed seasonal variation for total taxa abundance, whereas taxa richness showed seasonal variation for all sites. Taxa abundance was substantially lower at Sites 5 and 4. Site 5 constantly receives effluents from industries, so it is evident that change in hydrologic flow regime has not influenced pollution load in this site. A similar trend was observed at Site 4, which showed no seasonal variation. Site 4 is located just downstream of Site 5, which suggests that there was a slight water quality improvement at Site 4 and little to no seasonal variability with regard to anthropogenic litter. This supports the idea that macroinvertebrate assemblages in urban streams can be influenced by numerous factors such as flow regime, water quality, habitat availability, etc. [18,51].

Generally, changes in diversity and abundance of aquatic biota along the longitudinal gradient of rivers due to local environmental conditions have been widely observed [41,52]. The findings of this study have not deviated from this trend, and it is evident that the physical habitat quality changes along the longitudinal gradient of urban rivers do not vary from those observed in natural rivers. The Palmiet River has shown some resilience to anthropogenic disturbances and the ability to self-purify along the longitudinal gradient. Therefore, urban rivers should be considered comprehensive river systems as all ecological complexities resemble those of natural rivers.

### 4.4. Average Score Per Taxon

Macroinvertebrates as indicators of water quality have been used for decades, and they proved to be the most reliable pollution screening tool in both urban and natural rivers [45,49,53,54]. It is commonly known that most rivers harbor sensitive species in their headwaters where the water quality is relatively good [55]. A similar trend was observed in the present study, with a relatively high ASPT being observed in the headwaters. This was also supported by the NMDS plot, which showed clear discrimination for ASPT between Site 6 and other sites. Sensitive taxa such as amphipods, and palaemonids were only recorded in the headwaters, which suggests a good-quality water and macroinvertebrates habitat in this site. In contrast, Site 5 exhibited significantly lower ASPT, which coincided with the hypertrophic condition of the water in this site. A relatively low ASPT was recorded in rivers showing high concentrations of nutrients [56]. It is thus, reasonable to deduce that the poor water quality in this site might have driven the low ASPT. 

Nevertheless, the scores showed to improve as the river flows downstream, with Site 3 peaking again. This site was characterized by much-improved water quality and good quality habitat compared to Sites 4 and 5. According to Khudhair et al. [50] habitat quality is one of the crucial factors influencing macroinvertebrate assemblages. Sites 1 and 2 continued to exhibit better scores compared to Sites 4 and 5. This was expected as the habitat, and water quality in these sites were almost similar. These findings were comparable to those reported by Odume and Mgaba [47] in an urban river and Farrell et al. [57] in a river draining a catchment characterized by mining, industrial and agricultural activities. An ASPT has shown a clear negative relationship with NO_2_, NO_3_, NH_3_, and P. Therefore, the ASPT observed in the present study has successfully shown clear discrimination across sites with different water quality. It is evident that ASPT remains a more reliable tool to evaluate environmental conditions in an aquatic ecosystem compared to community structure and abundance.

## 5. Conclusions

Urban rivers are highly appreciated by urban dwellers, and their ecological integrity is of utmost importance [15]. This study has shown that the headwaters of Palmiet River is still in its pristine condition with a well-balanced macroinvertebrate assemblage. In contrast, the stretch within the industrial area was heavily polluted with relatively low diversity and an abundance of macroinvertebrates. The first site downstream of the industrial area (Site 4) exhibited a little improvement in water quality and macroinvertebrates diversity, with a considerable amount of anthropogenic litter providing refuge for macroinvertebrates. Sites 3 and 2 showed further improvement of water quality and macroinvertebrates diversity, whereas another increase in nutrient level was observed at Site 1. Despite nutrient level increase at Site 1, no decline of macroinvertebrates diversity was observed, which could be explained by the physical habitat quality. It is evident that the current condition of the river can still support biodiversity and the functioning of the river, but it is unclear if the system could endure further disturbance. These findings affirm the ecological importance of urban rivers as they provide refuge to aquatic biota. In some cases, anthropogenic litter was seen providing additional habitat for macroinvertebrates. Lastly, there is a Palmiet River rehabilitation project in place, so these findings may further provide a baseline for monitoring the improvement of the ecological conditions of this river system.

## Figures and Tables

**Table 1:** Invertebrates habitat assessment system scoring guidelines [23].

IHAS Score	Description	Ecological Category
>75	Excellent/Natural—Unmodified or almost natural conditions; natural biotic template will not be modified. Minimal risk or reduction in habitat availability.	A
65–75	Good—Largely natural with few modifications; only a small risk of modifying the natural biotic template. Risk to the availability of habitat moderate, availability of unique habitats at risk	B
55–64	Adequate/Fair—Modified state; moderate risk of modifying the biotic template occurs. Habitat unavailable to certain aquatic invertebrates.	C
<55	Poor—Largely modified unnatural state; large risk of modifying the biotic template. Natural required habitat generally unavailable to most aquatic invertebrates.	D

## Data Availability

The data are housed in Mendeley data https://doi.org/10.17632/stgy64rb9z.1 (accessed on 28 October 2021) and published in Data in Brief https://doi.org/10.1016/j.dib.2021.107493 (accessed on 25 October 2021).

## Appendix A

**Table A1:** A sheet for integrated habitat assessment score (adapted from McMillan 1998).

Integrated Habitat Assessment System (IHAS)						
River Name:						
Site Name:				Date:		
SAMPLING HABITAT	0	1	2	3	4	5
Stones in current (SIC)						
Total length (m) of broken water (riffles/rapids)	none	0–1	>1–2	>2–3	>3–5	>5
Total length (m) of submerged stones in current (run)	none	0–2	>2–5	>5–10	>10	
Number of separate SIC areas kicked	0	1	2–3	4–5	6+	
Average size (cm) of stones kicked (gravel < 2; bedrock > 20)	none	<2, >20	2–10	11–20	2–20	
Amount of stone surface clear (of algae, sediment, silt, etc.) (%)	n/a	0–25	26–50	51–75	>75	
Protocol: Time (mins) spent kicking SIC (gravel/bedrock = 0)	0	<1	>1–2	2	>2–3	>3
SIC Scores: (A = SIC boxes total; B = adjustment to equal 20; C = final total)	actual	A	adj.	B	max. 20	C
Vegetation						
Length (m) of fringing vegetation sampled (banks)	none	0–½	>½–1	>1–2	2	>2
Amount (m^2^) of aquatic vegetation/algae sampled	none	0–½	>½–1	>1		
Fringing vegetation sampled in:	none		run	pool		mix
Type of veg. (% leafy veg. vs. stems/shoots) (aq. veg. only = 49)	none	0	1–25	26–50	51–75	>75
Veg Scores: (D = Veg boxes total; E = adjustment to equal 15; F = final total)	actual	D	adj.	E	max. 15	F
Other habitats						
Stones Out Of Current (SOOC) sampled (m^2^) (protocol = 1 m^2^)	none	0–½	>½–1	1	>1	
Sand sampled (mins) (protocol = 1 min) (under = present below stones)	none	under	0–½	>½–1	1	>1
Mud sampled (mins) (protocol = ½ min) (under = present below stones)	none	under	0–½	½	>½	
Gravel sampled (mins) (protocol = ½ min) (if all, SIC stone size ≤ 2) *	none	0–½	½	>½ *		
Bedrock sampled (all = no SIC/sand/gravel) (if all, SIC stone size ≥ 20) *	none	some			all *	
Algal presence (1–2 m^2^ = algal bed; rocks = on rocks; isol. = isolated clumps)	>2 m^2^	rocks	1–2 m^2^	<1 m^2^	isol.	none
Tray identification (using time as per protocol)		under		correct		over
(* Note: SIC must still be filled)						
Other Habitat Scores:	actual	G	adj.	H	max. 20	I
(G = Other Habitat boxes total; H = adjustment to equal 20; I = final total)						
HABITAT TOTALS:			adj.	J	max. 55	K
(J = total adjustment [B + E + H]; K = Habitat Total [C + F + I])						
Stream condition						
Physical						
River make-up (2/3 mix = 2/3 types)	pool		run	rapid	2 mix	3 mix
Average stream width (m)		>10	>5–10	<1	02-Jan	>2–5
Average stream depth (m)	>2	>1–2	1	>½–1	½	<½
Approximate stream velocity (slow ≤ ½ m/s; fast ≥ 1 m/s)	still	slow	fast	med.		mix
Water color (discol. = discolored but still fairly clear)	silty	opaque		discol.		clear
Recent disturbances due to: (constr. = construction)	flood	fire	constr.	other		none
Bank/riparian vegetation is: (grass = incl. reeds; shrubs = incl. trees)	none		grass	shrubs	mix	
Surrounding impacts (erosn. = erosion/shear banks; farm = farmland)	erosn.	farm	trees	other		open
Anthropogenic litter	absent		similar		mix	
Anthropogenic litter effect			severe		none	
Left bank cover (%) (rocks and vegetation)	0–50	51–80	81–95	>95		
Right bank cover (%) (rocks and vegetation)	0–50	51–80	81–95	>95		
Stream Condition Total:					max. 45	L
Total IHAS Score: (K + L)					%	
